# Antibacterial and Cytotoxic Effects of Biosynthesized Zinc Oxide and Titanium Dioxide Nanoparticles

**DOI:** 10.3390/microorganisms11061363

**Published:** 2023-05-23

**Authors:** Samrin Habib, Farzana Rashid, Hunaiza Tahir, Iram Liaqat, Asma Abdul Latif, Sajida Naseem, Awais Khalid, Nazima Haider, Umme Hani, Rehab A. Dawoud, Yosra Modafer, Asia Bibi, Ohoud A. Jefri

**Affiliations:** 1 Department of Zoology, Lahore College for Women University, Lahore 54000, Pakistan; 2 Microbiology Laboratory, Department of Zoology, Government College University, Lahore 54000, Pakistan; 3 Department of Zoology, University of Education, Lower Mall Campus, Lahore 54000, Pakistan; 4 Department of Physics, Hazara University, Mansehra 21300, Pakistan; 5 Department of Pathology, College of Medicine, King Khalid University, Abha 61421, Saudi Arabia; 6 Department of Pharmaceutics, College of Pharmacy, King Khalid University, Abha 61421, Saudi Arabia; 7 Department of Biology, College of Science, Jazan University, Jazan 45142, Saudi Arabia; rdawood@jazanu.edu.sa (R.A.D.);; 8 Department of Zoology, The Women University, Multan 66000, Pakistan; 9 Department of Biological Science, Faculty of Science, King Abdulaziz University, Jeddah 21589, Saudi Arabia

**Keywords:** antibacterial, antioxidant, cytotoxicity, *Calotropis procera*, nanoparticles, TiO_2_, ZnO

## Abstract

Nanotechnology is a rapidly developing field of research that studies materials having dimensions of less than 100 nanometers. It is applicable in many areas of life sciences and medicine including skin care and personal hygiene, as these materials are the essential components of various cosmetics and sunscreens. The aim of the present study was to synthesize Zinc oxide (ZnO) and Titanium dioxide (TiO_2_) nanoparticles (NPs) by using *Calotropis procera* (*C. procera*) leaf extract. Green synthesized NPs were characterized by UV spectroscopy, Fourier transform infrared (FTIR), X-ray diffraction (XRD), and Scanning Electron Microscopy (SEM) to investigate their structure, size, and physical properties. The antibacterial and synergistic effects of ZnO and TiO_2_ NPs along with antibiotics were also observed against bacterial isolates. The antioxidant activity of synthesized NPs was analyzed by their α-diphenyl-β-picrylhydrazyl (DPPH) radical scavenging activity. In vivo toxic effects of the synthesized NPs were evaluated in albino mice at different doses (100, 200, and 300 mg/kg body weight) of ZnO and TiO_2_ NPs administered orally for 7, 14, and 21 days. The antibacterial results showed that the zone of inhibition (ZOI) was increased in a concentration-dependent manner. Among the bacterial strains, *Staphylococcus aureus* showed the highest ZOI, i.e., 17 and 14 mm against ZnO and TiO_2_ NPs, respectively, while *Escherichia coli* showed the lowest ZOI, i.e., 12 and 10 mm, respectively. Therefore, ZnO NPs are potent antibacterial agents compared to TiO_2_ NPs. Both NPs showed synergistic effects with antibiotics (ciprofloxacin and imipenem). Moreover, the DPPH activity showed that ZnO and TiO_2_ NPs have significantly (*p* > 0.05) higher antioxidant activity, i.e., 53% and 58.7%, respectively, which indicated that TiO_2_ has good antioxidant potential compared to ZnO NPs. However, the histological changes after exposure to different doses of ZnO and TiO_2_ NPs showed toxicity-related changes in the structure of the kidney compared to the control group. The current study provided valuable information about the antibacterial, antioxidant, and toxicity impacts of green synthesized ZnO and TiO_2_ NPs, which can be influential in the further study of their eco-toxicological effects.

## 1. Introduction

For the advancement of science, nanotechnology is a rapidly expanding and diverse field of study that focuses on the synthesis, characterization, strategy, and manipulation of particle structures between 1 and 100 nm in size [1]. They are commonly used in a variety of fields including material science, food processing, agriculture, cosmetics, diagnostics, and medicine [2]. Due to its extraordinary physico-chemical and morphological characteristics, it is also being employed in numerous sectors, including biomedicine, bio sensing, MRI, targeted drug administration, phytopathology, and agriculture biotechnology [3].

Metal and metal oxide NPs are usually used in cosmetology and dermatology, particularly for the treatment of bacterial and fungal skin infections, and sun protection and scar reduction creams that speed up skin cell repair. The use of NPs in dermatology and skin care products has enhanced patients’ quality of life [4].

In the medical world, successfully treating bacterial infections has become a major concern. Bacterial infections are a leading cause of long-term illnesses and death. Because of their cost-effectiveness and potent results, antibiotics have been utilized to treat bacterial illnesses. For many years, antibiotic therapy has been critical in fighting against microbial infections [5]. Previous studies, on the other hand, have suggested that widespread antibiotic use has resulted in the creation of multidrug-resistant bacterial strains. Further, antibiotic overuse has recently resulted in the development of super-bugs that are resistant to practically all antibiotics [6]. Prospective antibacterial agents to which microorganisms may not build resistance include metals and metal oxide NPs [7,8].

NPs can be prepared via physical, chemical, and biological methods. In the chemical synthesis and stabilization of NPs, chemicals are used which are toxic to human health and which produce unfriendly byproducts [9]. NP size, concentration, and surface charge can influence agglomerate formation. They cross the bacterial cell wall and release ions or may remain deposited in the cell. Due to their very small size, they can easily pass through the peptidoglycans layer of the cell wall, hence damaging the cellular membrane, disturbing the cellular metabolism, generating reactive oxygen species (oxidative stress), and inhibiting transcription. The ions released by the NPs interact with sulfur and phosphorus molecules that are present in the proteins associated with the cell wall and membrane, producing multiple pores and resulting in the outflow of intracellular content. This causes ion imbalance, allowing ions to pass into the cytoplasm through the plasma membrane; ZnO and TiO_2_ are the two nanomaterials that have been studied the most frequently among these, and they exhibit antibacterial activity and toxicity to various organisms and cell lines. ZnO and TiO_2_ NPs interact with the cell membrane and release free radicals that cause cell membrane distortion and the degradation of proteins [10,11,12,13,14].

Chemical methods that have been used in the past have been linked to bio-accumulation and the release of harmful materials into the environment. As well, the toxicity issue with living cells restricts their use in the medical field. Green synthesis is regarded as a safe, accurate, and dependable method of NPs fabrication. NPs made by biological methods are favored over those made by physical and chemical methods because of their specific properties [15]. Compared to chemical approaches, biological techniques using microbes and plants, as well as plant extracts, have been suggested for metal NPs. Many biological systems, including fungi, yeast, and bacteria, have been utilized in the production of NPs [16].

A cultivable wild xerophytic shrub called *C. procera* (subfamily: Asclepiadaceae) is found in South America, Asia, and Africa. The milky white latex produced by *C. procera* has a number of medicinal, antiviral, antibacterial, and antioxidant benefits [17]. For the biological synthesis of NPs, we preferred *C. procera* because it is a multipurpose plant, is easily available, and is used as a therapeutic substance in various countries, including North Africa, South Asia, and others, for the prevention and treatment of many ailments [18]. *C. procera* has been used as a folk medicine for many years. The plant is reported as effective in treating skin, digestive, respiratory, circulatory, and neurological disorders. This plant was also used to treat fevers, elephantiasis, nausea, vomiting, diarrhea, and cancer [19]. In light of *C. procera*’s significant pharmacological role, we anticipated that using this particular plant extract as a reducing agent would make it possible to cap NPs with vital phytochemicals.

ZnO is a substance that has both semiconducting and piezoelectric capabilities, as well as being safe and biocompatible. The many favorable features of ZnO, such as significant excitation energy, etc., at ambient temperature, are attracting a large amount of attention [20,21]. Due to their numerous uses in photocatalysis, antimicrobial defense, and water purification, ZnO-NPs are quite important. They exhibit characteristics that set them apart from conventional NPs. Moreover, NPs are used in the cosmetics sector to create sunscreen lotions that protect the human body from ultraviolet rays [22]. Because of its unique optical, electrical, and chemical properties, ZnO has recently attained a large amount of attention in terms of potential electronic applications. ZnO solution shows less antimicrobial activity as compared to ZnO NPs that have potent antibacterial properties against a wide range of pathogenic bacteria [23].

TiO_2_ has been used in a variety of industrial and cosmetic applications. As a result, it may be employed in a variety of fields, including photocatalysis, antimicrobial products, lithium-ion battery electrodes, and super capacitors. Furthermore, TiO_2_ offers appealing properties because of its non-flammability and non-toxicity, as well as its high relative abundance, corrosion resistance, low cost, increased safety, and being eco-friendly [24].

The present study is designed to synthesize and characterize ZnO and TiO_2_ NPs by utilizing the bio components of *C. procera* leaf extract and the in vitro investigation of their cytotoxicity and antibacterial activities against bacteria causing skin infections, which will provide information about the applications of synthesized NPs in dermatology. UV spectroscopy, FTIR, XRD, and SEM were performed to charaterize the green synthesized ZnO and TiO_2_ NPs. In addition, antioxidant potential followed by toxicity assessment was also carried out. Furthermore, no comparative studies have been performed on green synthesized ZnO and TiO_2_ NPs by using *C. procera* leaf extract. This study provides a comprehensive approach to the possible biomedical capability of green synthesized NPs.

## 2. Materials and Methods

### 2.1. Materials

*C. procera* leaves were collected from Lahore for the preparation of extract, and zinc acetate dehydrate Zn(CH_3_COO)_2_·2H_2_O (Sigma-Aldrich, Gillingham, UK) and titanium tetra iso-propoxide [Ti (OCH (CH₂)] (Sigma Aldrich) were used as precursors. Bacterial culture media such as Nutrient agar, Luria bertani (lb) broth, and Muelle–Hhinton agar (Sigma-Aldrich) were utilized to test the antibacterial activity of ZnO and TiO_2_ NPs. The antibiotics imipenem (10 µg) and ciprofloxacin (5 µg) (BioAnalyse Ltd. Ankara, Turkey) were used in this study. Concentrations of NPs were made in DMSO. Study was carried out from September 2021 to December 2022.

Chemically synthesized and characterized ZnO NPs were already availiable in lab as reported in our previous study [23]. Likewise, Chemical TiO_2_ NPs were also characterized previously and kindly provided by Irshad et al. [24].

### 2.2. Plant Extraction Preparation

Fresh leaves of *C. procera* (Sodom apple) were collected and repeatedly cleaned thoroughly with distilled-deionized water, then dried to remove the residual moisture. *C. procera* leaf extract was prepared by placing about 50 g of dried, finely cut leaves into a 250 cm^3^ beaker containing 100 cm^3^ deionized water and the mixture was boiled for 60 min at 70 degree Celsius (°C) until the color of the aqueous solution turned light yellow; then the solution was allowed to cool. The extract obtained was filtered through Whatman No. 1 filter paper and stored at room temperature to be used for further experiments.

### 2.3. Green Synthesis of ZnO and TiO_2_ NPs with C. procera Leaf Extract

#### 2.3.1. Synthesis of ZnO NPs

For the synthesis of ZnO NPs, 50 mL leaf extract of *C. procera* was taken and boiled at 60–70 °C using magnetic stirrer. As the temperature reached 60 °C, 5 g of zinc acetate dehydrate was added into the solution of plant extract. The mixture was heated until it turned into a thick paste. This paste was collected in ceramic crucible and heated at 400 °C for 2 h in an air-heated furnace [25]. A light-yellow powder was acquired. This was carefully collected and ground in pestle mortar to obtain a fine powder for further use.

#### 2.3.2. Green Synthesis of TiO_2_ NPs

*C. procera* leaf extract and titanium isopropoxide were mixed in a volumetric ratio 1:3 and were added into 300 mL distilled water. The mixture was then stirred at room temperature for 2 h. Thereafter, the mixtures were sonicated for 30 min and calcined at 300 °C, then filtered by using filter paper. The filtrate was allowed to dry at room temperature [26]. Afterwards, the dried material was collected and ground in mortar pestle to obtain fine NPs.

### 2.4. Characterization of ZnO and TiO_2_ NPs

The synthesized ZnO and TiO_2_ NPs were characterized by using a combination of spectroscopic techniques such as UV-visible spectroscopy (UV-Vis; AE-S70-1U; AELAB GUANGZHOU Co., Ltd., Guangzhou, China, A & E Lab Instruments (Guangzhou) Co., Ltd., Guangzhou, China), X-ray diffraction (XRD D8 discover, Bruker, Germany), Fourier transform infrared spectroscopy [FTIR; (P/N 206-72010, Tracer SHIMADZU, Kyoto, Japan)] and Scanning Electron Microscopy (SEM; Evo LS10 Zeiss, Jena, Germany) to analyze their crystallinity, structure, composition, and size. ImageJ (https://imagej.net/ij/, accessed on 21 August 2022) was used to determine the size of synthesized NPs.

UV-visible spectrometer was used to investigate the optical characteristics of the NPs. Band gap energy of the ZnO and TiO_2_ NPs was measured by the formula:

E = h·c/*λ*

where h, c, and λ represent Planck’s constant, velocity of light, and wavelength of maximum absorption, respectively. ZnO and TiO_2_ NPs band gap energy was estimated at 3.17 eV and 4.1 eV, respectively, by putting the value in the above-mentioned equation.

X-ray diffraction was used to find the structure and crystalline size of the NPs. The average size of the ZnO and TiO_2_ NPs was calculated by using Debye-Scherrer’s formula:

L = Kλ/β·cosθ

where L is the crystalline size of the NPs, K is the Scherrer constant, and λ, β, and θ represents the wavelength of X-ray, FWMH in radians, and Brags’ angle, respectively.

The average crystalline sizes of the ZnO and TiO_2_ were calculated by using the above formula as 10.11 and 18.708 (nm), respectively.

### 2.5. Bacterial Strains

Non-repetitive thirty (30) clinical isolates from skin exudates such as pus, wounds, burn, etc., were collected from microbiology laboratory of services hospital and general hospital of Lahore and characterized by morphological and biochemical tests, i.e., citrate and catalase test using 16S rRNA sequencing [27]. The selected strains were *Staphylococcus aureus* (*S. aureus*; MT448672), *Streptococcus* sp., *Escherichia coli* (*E. coli*; MT448673), and *Pseudomonas aeruginosa* (*P. aeruginosa*; MN900691). These selected bacterial strains were sensitive to imipenem and resistant to ciprofloxacin.

### 2.6. Antibacterial Activity of ZnO and TiO_2_ NPs

Antibacterial activity of *C. procera* leaf extract, and ZnO and TiO_2_ NPs, was determined against Gram negative and Gram positive bacteria using various concentrations (10, 20, 30, and 40 mg/mL) of green synthesized ZnO and TiO_2_ NPs and using plant extract, following Kirby-Bauer disc diffusion method [27]. The antibacterial tests were performed in the light, including UV. Isolated strains were subcultured in nutrient broth at 30 °C for 24 h. Sterilized standard filter paper discs were soaked in different concentrations of ZnO and TiO_2_ NPs. Blank disc saturated with distilled water was used as negative control and antibiotics [imipenem and ciprofloxacin] were used as positive controls. Plant extract and chemically synthesized NPs were also tested in parallel as controls. The experiment was performed in triplicate and ZOI was measured.

### 2.7. Synergistic Effects of NPs with Antibiotics against Clinical Isolates

The synergistic effects of ZnO and TiO_2_ NPs with antibiotics, i.e., imipenem and ciprofloxacin were checked against bacterial pathogens collected from human skin infections as described by Liaqat et al. [28]. Mueller–Hinton agar was prepared and poured into pre-labeled sterilized petri plates. After solidification of these plates, freshly prepared cultures of overnight grown bacterial strains were swabbed and ZOI was measured after 24 h. Appropriate controls were run in parallel.

### 2.8. Antioxidant Potential of ZnO and TiO_2_ NPs by DPPH Method

The antioxidant potentials of ZnO and TiO_2_ NPs were evaluated by DPPH (1,1-diphenyl-2-picryl-hydrazyl, Sigma-Aldrich, Darmstadt, Germany) radical scavenging assay as described by Dridi [29] with little modifications. The experiment was performed in triplicates. The L-ascorbic acid was used as control by dissolving 1 mg in 1 mL methanol. Then, 1 mL of each sample concentration (0, 10, 20, 30, and 40 mg/mL) was mixed with 2 mL of DPPH solution (1:2) and shaken well. The solution was incubated for 30 min in the dark at 37 °C. The absorbance was measured at 517 nm using spectrophotometer.

The scavenging activity % of ZnO and TiO_2_NPs was evaluated via equation:

$$\%\;\mathrm{scavenging}\;\mathrm{activity}=\frac{\left(Asorbance\;of\;control-Absorbance\;of\;sample\right)}{Absorbance\;of\;control}\times 100\%$$

### 2.9. In Vivo Study

#### 2.9.1. Ethical Approval

The experiments were executed in agreement with approval obtained from the Bio ethics committee of Government College University, Lahore via No. GCU-IIB-497 dated 21 January 2021.

#### 2.9.2. Experimental Animals and Toxicity Evaluation of Green Synthesized ZnO and TiO_2_ Nanoparticles

The toxic effects of green synthesized ZnO and TiO_2_ NPs were studied in vivo. For this purpose, forty two (42) 7–8 week-old healthy mice were purchased from the University of Veterinary and Animal Sciences (UVAS). Animals weighing 25–30 g were acclimatized for 7 days and provided standard conditions such as 12 h light/dark cycle and 25 ± 5 °C. They were fed standard diet throughout the experiment and allowed a pellet diet and to drink water *ad libitum*. Experiments were performed in triplicate as described by Noori [30].

In brief, animals were divided into three groups: one control group and two experimental groups. Each experimental group was further divided into three groups on the bases of dose given to the animals (each group comprised 6 mice). Control group was divided into two subgroups: Feed and saline solution were given to the control subgroup 1 and plant extract was given to the 2nd control subgroup (03 mice each). Low (100 mg/kg bw), medium (200 mg/kg bw), and high doses (300 mg/kg bw) of ZnO and TiO_2_ NPs were given orally to the experimental groups for 7, 14, and 21 days using gavage.

#### 2.9.3. Histological Examinations

For histological studies, the animals’ kidneys, livers, and organs were taken and fixed in 10% formalin. Next, slides were prepared and stained with hematoxylin and eosin (H and E). The slides were observed under camera-fitted microscope at 40×. Considering the absence of any histological alteration in control mice, only one of them was considered for result discussion Also, the current study describes the findings of kidney histology only.

### 2.10. Statistical Analysis

All the experiments, including those for antibacterial, antioxidant, and cytotoxic activity, were carried out in triplicate. The data were evaluated by means ± standard deviation using Microsoft Excel version (2013). Significant differences between antimicrobial activities of both bio-synthesized ZnO and TiO_2_ NPs at various concentrations were analyzed by IBM SPSS (version 25). The level of significance was considered at *p* ≤ 0.05.

## 3. Results

The observed change in color of Zn (yellow) and Ti solution (pink) was taken as an affirmation of the reduction of zinc acetate dehydrate and titanium tetra iso-propoxide salt into ZnO and TiO_2_ NPs, respectively.

### 3.1. Characterizations of ZnO and TiO_2_ NPs

#### 3.1.1. UV-Visible Spectroscopy

**Figure 1:**
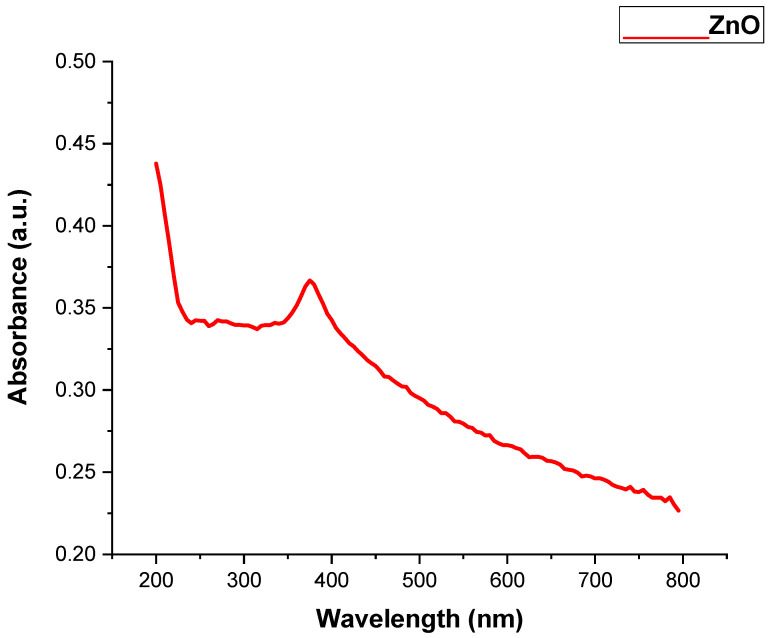
UV Visible Spectra of ZnO NPs.

**Figure 2:**
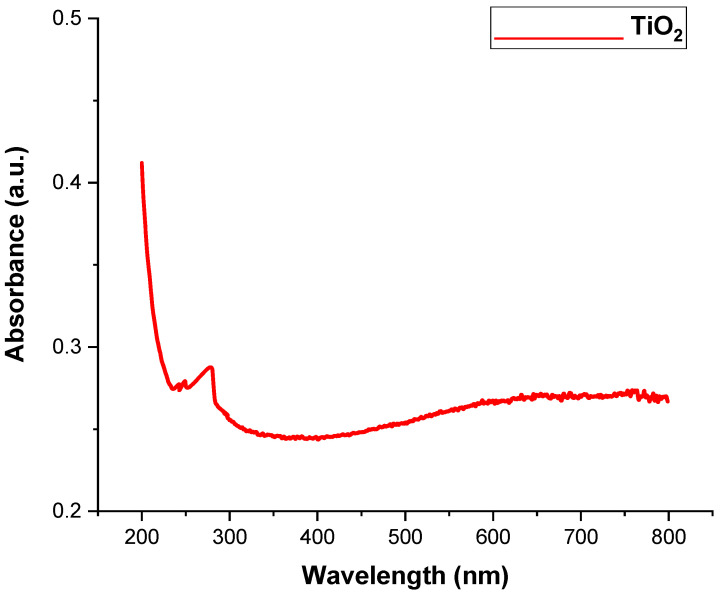
UV Visible Spectra of TiO_2_ NPs.

The absorption spectrum of the ZnO and TiO_2_ NPs is represented in Figure 1 and Figure 2, and ranged from 200 to 800 nm. The ZnO and TiO_2_ NPs showed absorption bands at 390 nm and 295 nm, respectively. Similar absorption peaks were noted after 24, 48, 72, 96, and 120 h, confirming their stability. The purity of the ZnO and TiO_2_ NPs was determined by the absence of any peak in the range of the absorption spectrum.

#### 3.1.2. X-ray Diffraction

**Figure 3:**
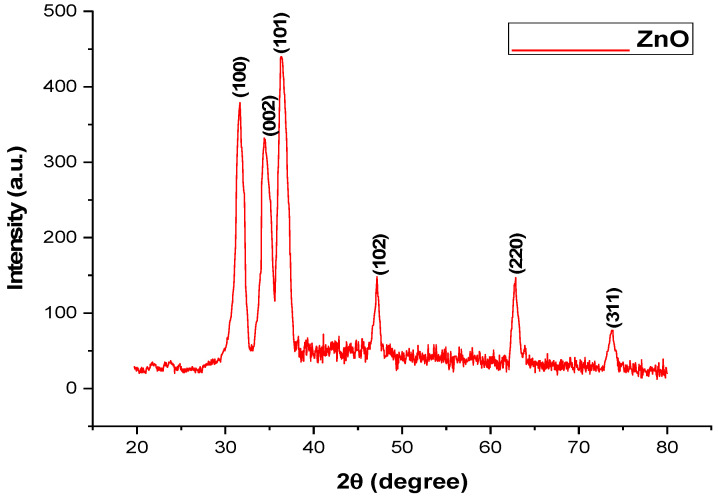
X-ray Diffraction of green synthesized ZnO NPs.

**Figure 4:**
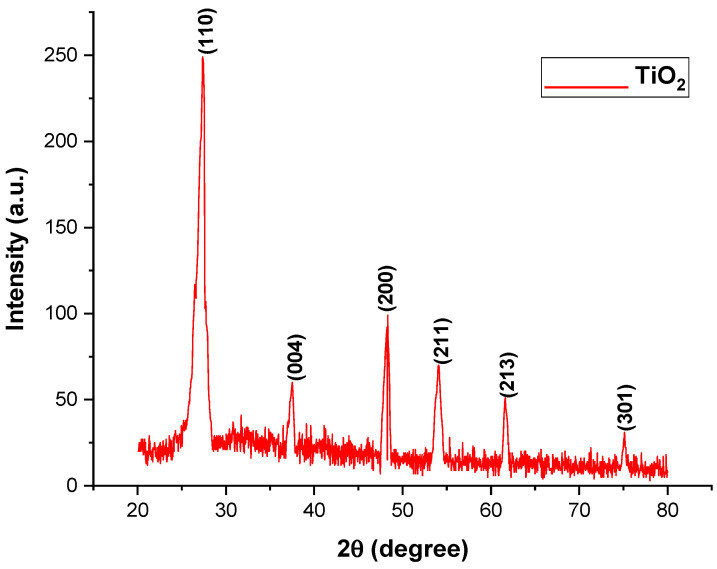
X-ray Diffraction of green synthesized TiO_2_ NPs.

The range of the ZnO and TiO_2_ XRD pattern lies between the diffraction angles 20 < 2θ < 80 as shown in Figure 3 and Figure 4. In Figure 3, ZnO NPs showed the highest orientation peak at 2θ = 36.27°, with other peaks at 31.67°, 34.43°, 47.53°, 62.97°, and 73.42°, along with diffraction index values of (100), (002), (101), (102), (220), and (311), respectively (JCPDS card number 5-0566). On the other hand, Figure 4 represents different peaks of TiO_2_ at the angles of 27.52°, 37.36°, 48°, 54.75°, 62.19°, and 74°, corresponding to (110), (004), (200), (211), (213), and (301), with good agreement with the JCPDS card (21-1272). The purity of ZnO and TiO_2_ NPs was confirmed by the location of the peak and the observation of no extra peak in the diffraction pattern. The size of the NPs was correlated with the width of the peak. An increase in peak width led to a decreased size of the NPs.

#### 3.1.3. FT-IR Spectroscopic Analysis of the ZnO and TiO_2_ NPs

**Figure 5:**
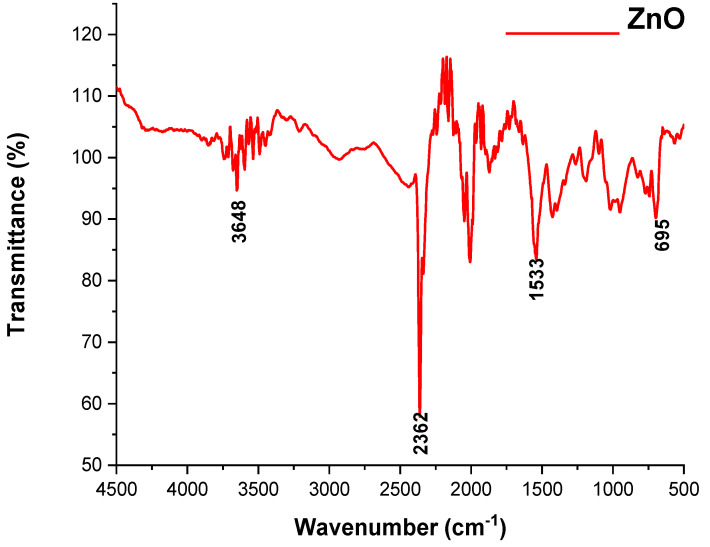
FTIR spectra of ZnO NPs.

**Figure 6:**
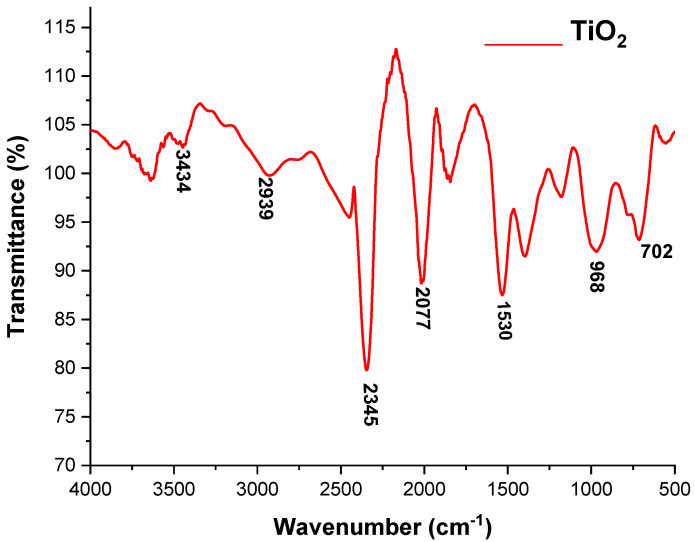
FTIR spectra of TiO_2_ NPs.

FTIR is used to record the optical response of molecular species, which first manifests as vibrations and then absorbs the IR signal, as shown in Figure 5 and Figure 6. ZnO and TiO_2_ NPs were synthesized using leaves of *C. Procera*, and their FT-IR spectra in 100% transmittance mode ranged from 4000 to 500 cm^−1^, as shown in Figure 5 and Figure 6.

According to the FT-IR spectrum of the ZnO NPs, the peak formed in the lowest region, 500–700 cm^−1^, which indicated the formation of ZnO NPs. The highest peaks, at 1533, 2362, and 3648 cm^−1^, indicated the formation of an O-H bond. The formation of the TiO_2_ NPs was determined by the peak appearing at 702 and 968 cm^−1^. The band observed at 1530 cm^−1^ was linked to NPs having absorbed airborne water molecules during analysis. The band that appeared at 3431 cm^−1^ confirmed that the produced TiO_2_ NPs had a hydroxyl group. All of these spectrum peaks in both the ZnO and TiO_2_ NPs indicated the presence of secondary metabolites such as alkaloids, peptides, terpenoids, polyphenols, and flavonoids attached to these NPs; therefore, these secondary metabolites may be responsible for the formation of ZnO and TiO_2_ NPs.

#### 3.1.4. Scanning Electron Microscopy

**Figure 7:**
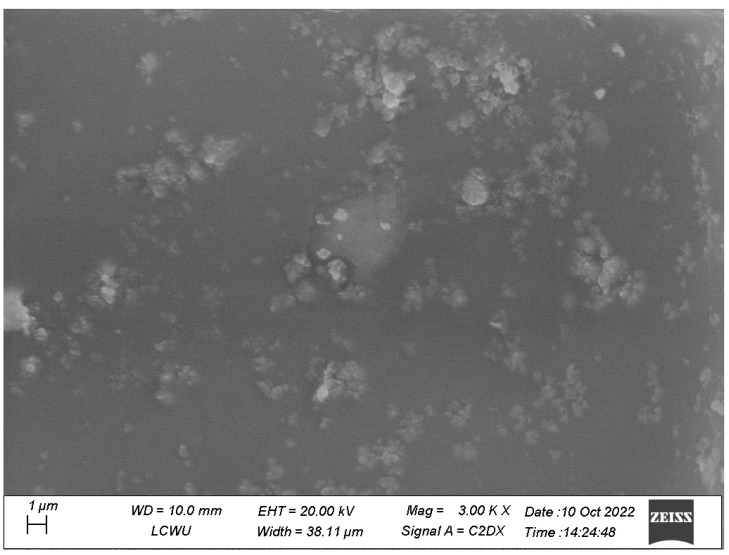
SEM micrograph of biosynthesized ZnO NPs.

**Figure 8:**
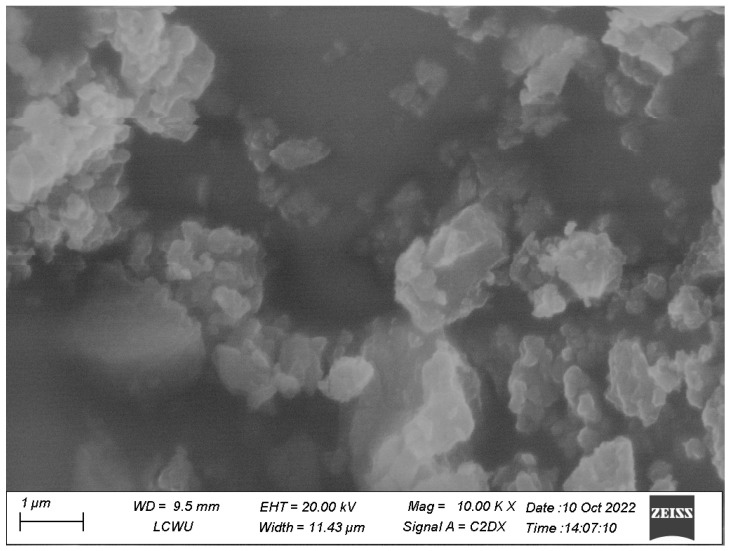
SEM micrograph of biosynthesized TiO_2_ NPs.

The size and morphology of the ZnO and TiO_2_ NPs were depicted using SEM, as shown in Figure 7 and Figure 8, respectively. SEM micrographs showed the individual and number of aggregates. The data revealed that spherical-shape ZnO NPs and irregular-shape TiO_2_ NPs were formed with diameter ranges of 10–35 nm. Figure 7 and Figure 8 show that smaller-size ZnO and TiO_2_ NPs were formed with an average size of 15 and 18 nm, respectively.

### 3.2. Antibacterial and Synergistic Effects of Green Synthesized ZnO and TiO_2_ NPs

#### 3.2.1. Antibacterial Activity and Synergistic Effects of ZnO NPs against Bacterial Strains

**Figure 9:**
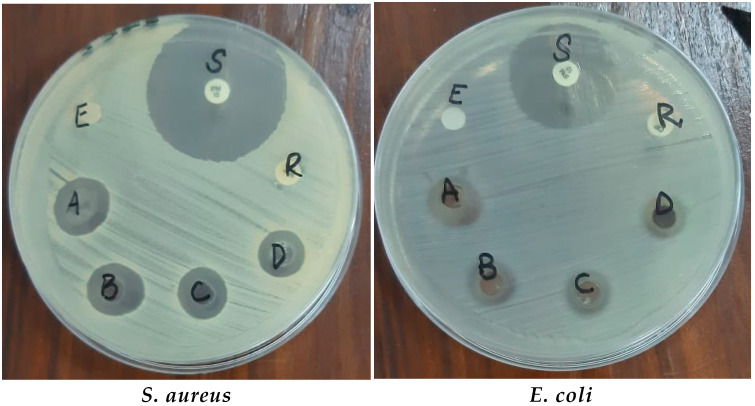
Antibacterial activity of ZnO NPs against bacterial strains. Where: A: 40 mg/mL of ZnO, B: 30 mg/mL of ZnO, C: 20 mg/mL of ZnO, D: 10 mg/mL ZnO, E: Negative Control (autoclave distilled Water), S: bacteria sensitive to antibiotics (imipenem), and R: bacteria resistant to antibiotic (ciprofloxacin).

The antibacterial activity and synergistic effects of green synthesized ZnO NPs were tested by disc diffusion assay against different Gram positive and negative bacterial strains. When different concentrations (10, 20, 30, and 40 mg/mL) of ZnO NPs were tested, the ZOIs were formed alone and along with antibiotics, as shown in Figure 9 and Table 1.

#### 3.2.2. Antibacterial Activity and Synergistic Effects of TiO_2_ NPs against Bacterial Strains

**Figure 10:**
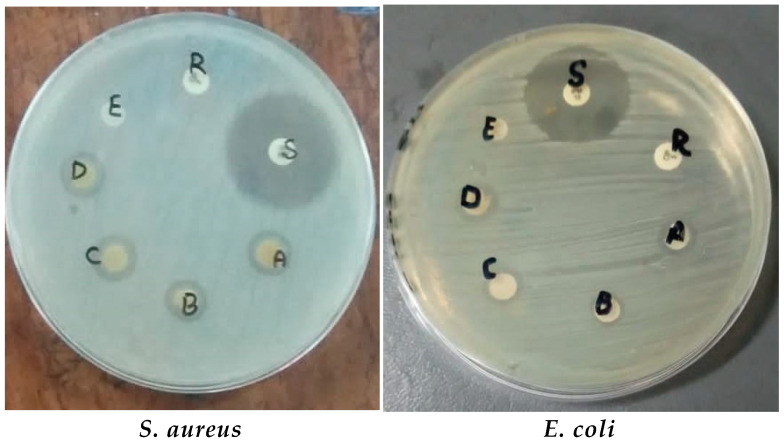
Antimicrobial activity of TiO_2_ NPs. Where: A: 40 mg/mL of TiO_2_, B: 30 mg/mL of TiO_2_, C: 20 mg/mL of TiO_2_, D: 10 mg/mL TiO_2_, E: Negative Control (autoclave distilled Water), S: bacteria sensitive to antibiotics (imipenem), and R: bacteria resistant to antibiotic (ciprofloxacin).

The effects of TiO_2_NPs alone and combined with antibiotics at different concentrations (10 mg/mL, 20 mg/mL, 30 mg/mL, and 40 mg/mL) in DMSO were checked against both Gram negative and Gram positive bacterial strains. The highest ZOI were obtained at a 40 mg/mL concentration of the NPs. Chemically synthesized TiO_2_ NPs showed ZOI in the range of 5–7 mm. The antibacterial effects of NPs are shown in Figure 10 and Table 2.

The results indicated that, among the bacterial strains, *S. aureus* showed the highest ZOI of 14 mm and *E. coli* showed a minimum ZOI of 10 mm at 40 mg/mL conc. of TiO_2_ NPs. Chemically synthesized TiO_2_ NPs showed ZOIs in the range of 5–7 mm. ZOIs formed at various concentrations were increased in combination with antibiotics, as described in Table 2.

### 3.3. Antioxidant Activity of ZnO and TiO_2_ NPs by DPPH Assay

**Figure 11:**
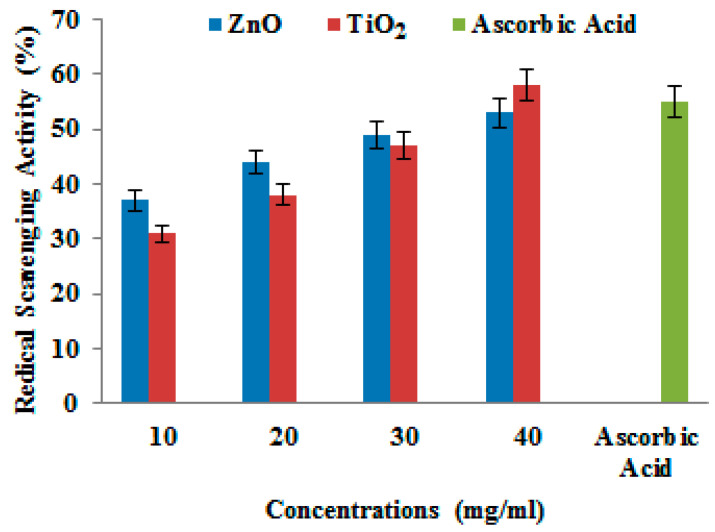
Radical scavenging activity of different concentrations of ZnO and TiO_2_ NPs.

The decrease in absorbance at the 517 nm wavelength indicated a reduction in DPPH. The results are shown in Figure 11. Different concentrations, i.e., (10 mg/mL, 20 mg/mL, 30 mg/mL, and 40 mg/mL) of ZnO and TiO_2_ NPs were used to measure the antioxidant activity by spectrophotometer. The DPPH activity showed that the free radical percentage scavenging potential of ZnO NPs and TiO_2_ was 53% and 58.7% at 40mg/mL, respectively. Increased concentration of NPs also increased the scavenging potential compared with ascorbic acid (55.5 ± 0.001).

### 3.4. Effects of ZnO and TiO_2_ NPs on the Kidney

#### Histological Examination

**Figure 12:**
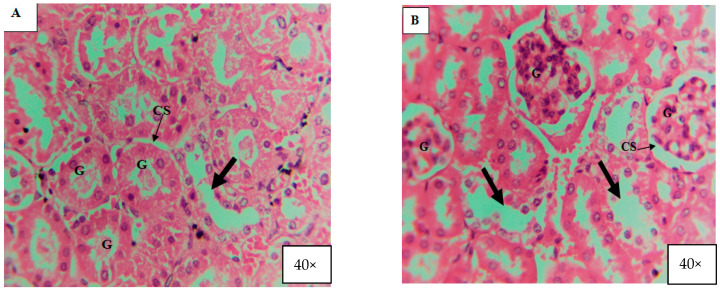

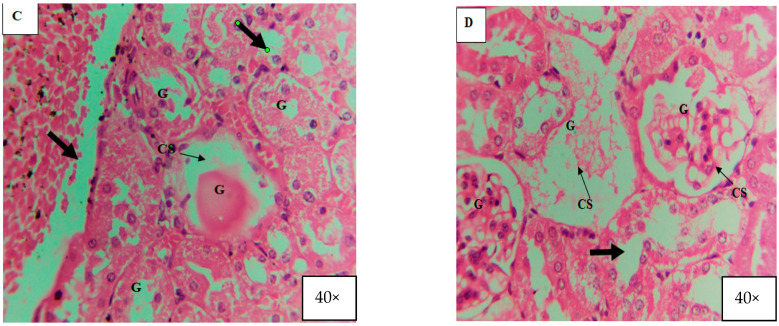
Histology of mice kidney treated with ZnO NPs (40X) after 21 days. (**A**) = Section of the kidney from the control group showing normal glomeruli structure (G), renal capsular space (CS), and normal cellular space (thick arrow). (**B**) = Section of the kidney treated with 100 mg/kg (low dose) concentration of ZnO NPs for 21 continuous days, showing distortion or shrinkage of glomeruli (G) structure, increase in capsular space (CS), and also increase in cellular space (thick arrow). (**C**,**D**) Section of the kidney treated with 200 mg/kg (medium dose) and 300 mg/kg (High Dose) concentration of ZnO NPs for 21 continuous days, showing distortion and swelling of glomeruli (G) structure, increase in capsular space (CS), and also increase in cellular space (thick arrow) and edema formation (cells wash out).

**Figure 13:**
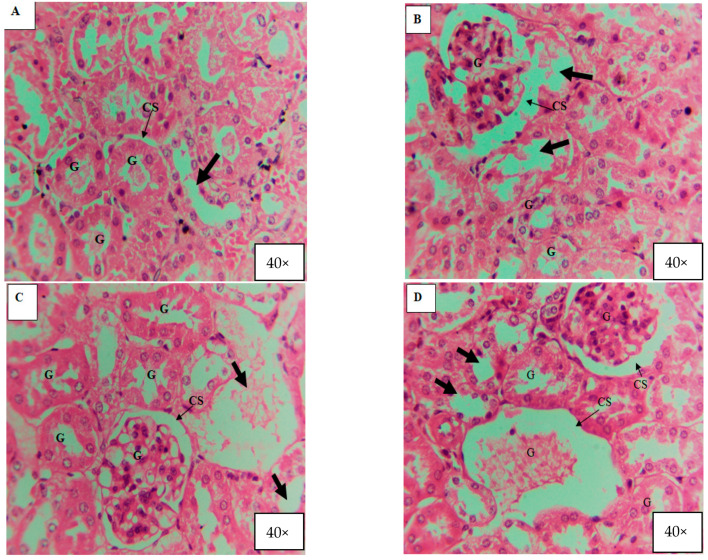
Histology of mice kidney after 21 days treated with TiO_2_ NPs (40×). (**A**) Section of the kidney from the control group showing normal glomeruli structure (G), renal capsular space (CS), and normal cellular space (thick arrow). (**B**) Section of the kidney treated with 100 mg/kg (low dose) concentration of TiO_2_ NPs for 21 continuous days, showing distortion or shrinkage of glomeruli (G) structure, increase in capsular space (CS), and also increase in cellular space (thick arrow). (**C**,**D**) Section of the kidney treated with 200 mg/kg (medium dose) and 300 mg/kg (High Dose) concentration of TiO_2_ NPs for 21 continuous days, showing distortion and swelling of glomeruli (G) structure, increase in capsular space (CS), and also increase in cellular space (thick arrow) and edema formation (cells wash out).

According to the current investigation, after 7, 14, and 21 days of exposure, kidney tissues underwent alterations caused by ZnO and TiO_2_ NPs at concentrations of 100 mg/kg, 200 mg/kg, and 300 mg/kg body weight. It was observed that smaller-size (about 20 nm) NPs directly affected the kidney tissue while larger-size NPs accumulated in the liver. A normal renal cortex and normal nephritic tubules were visible in the control group’s kidney sections (Figure 12A and Figure 13A). In contrast to the control group, the second group, receiving treatment with 100 mg/kg body weight/day displayed pathological abnormalities, such as: the medulla showed a small expansion in the collecting tubules while the renal cortex was normal (Figure 12B and Figure 13B). Inflammatory cells, in particular mononuclear cells, gathered around the blood vessels and between renal tubules in the kidney in the third group, which received treatment with 200 mg/kg body weight/day ZnO and TiO_2_ NPs (Figure 12C and Figure 13C). The fourth group that received treatment with 300 mg/kg displayed more toxic effects, including cell destruction, increased intracellular spaces, and distorted glomeruli (Figure 12D and Figure 13D).

## 4. Discussion

The production of novel antimicrobial agents led to the development of nanotechnology. ZnO and TiO_2_ NPs have received significant attention among all metal oxide NPs due to their versatility and resistance to extreme environments [31,32]. The present research work was designed to synthesize the ZnO and TiO_2_ NPs using *C. procera* leaf extract. Preliminary phytochemical analysis indicated that *C. procera* possess the phenolic compounds tannins, steroids, alkaloids, saponins, glycosides, terpenoids, proteins, flavonoids, coumarins, and anthraquinones in greater concentrations in the ethyl acetate and sterile distilled water extracts [33]. *C. procera* synthesized ZnO and TiO_2_ NPs act as the best reducing and stabilizing agents. The current study focused on synthesis and characterization, along with antibacterial potential and toxicity evaluation of ZnO and TiO_2_ NPs.

UV-visible spectroscopy is a reliable technique to obtain information about the absorbance spectra of NPs. Both the ZnO and TiO_2_ NPs showed UV-vis absorption bands at 390 and 295 nm, respectively. The particular peak for TiO_2_ NPs at 295 nm showed the formation of TiO_2_ NPs and is in accordance with previously published data by Irshad et al. [24]. Minor variations in the absorption peak at different wavelengths may be attributed to the sensitivity of the UV spectrum to many factors such as the shape, size, and agglomeration of the particles. Furthermore, similar peaks via UV-Vis spectrophotometer after 24, 48, 72, 96, and 120 h of both NPs confirmed their stability [34]. Another characterization tool used was XRD. XRD peaks are produced by constructively interfering with a monochromatic beam of X-rays, dispersed at particular angles from each set of lattice planes in a sample. As a result, the X-ray diffraction pattern represents a material’s unique signature of periodic atomic groupings [34]. The X-ray diffraction results showed that the range of the ZnO and TiO_2_ XRD patterns lie between the diffraction angles 20 < 2θ < 80. ZnO NPs showed the highest orientation peak at 2θ = 36.27°, with other diffraction peaks at 31.67°, 34.43°, 47.53°, 62.97°, and 73.42°, along with diffraction index values, and were keenly indexed as the cubic phase of ZnO and related to the diffraction planes of ZnO, (100), (002), (101), (102), (220), and (311), respectively (JCPDS card number 5-0566). In the case of TiO_2_, the angle values were shown to have a mixture between anatase and rutile, the peak from 27 degrees belonged to the rutile phase and was observed to be the principal peak. The same was true for 54 degrees, making it rutile.

FTIR is also an important tool for the determination of chemical bonds present in molecules [35]. The structure, morphology, and composition of the ZnO and TiO_2_ NPs were analyzed by the spectrum peak position and absorption spectrum band [36]. The FT-IR spectrum of ZnO NPs showed the peak formation in the lowest region (500–700 cm^−1^). A previous study described that an absorbance peak at 500 cm^−1^ alludes to Zn-O, which is similar to our result. The highest peaks at 1533 cm^−1^ indicated -C=C- stretching compared to O-H bond stretching at 3648 cm^−1^ [37]. Formation of the TiO_2_ NPs was determined by the peak appearing at 702 and 968 cm^−1^. The band observed at 1530 cm^−1^ was linked to NPs having absorbed airborne water molecules during analysis. The band that appeared at 3431 cm^−1^ confirmed that the produced TiO_2_ NPs had a hydroxyl group. All of these spectrum peaks in both ZnO and TiO_2_ NPs indicated the presence of secondary metabolites such as alkaloids, peptides, terpenoids, polyphenols, and flavonoids, which may have participated in the formation of ZnO and TiO_2_ NPs.

SEM showed the individual and number of aggregates by formed NPs. The images revealed spherical shape ZnO and irregular shape TiO_2_ NPs with diameters of 10–35 nm. Chemically synthesized ZnO NPs were reported to be cubic compared to TiO_2_ NPs [23,24]. An average size of 15 and 18 nm for both ZnO and TiO_2_ NPs was observed, respectively. The presence of different secondary metabolites such as alkaloids, peptides, terpenoids, polyphenols, and flavonoids cause NPs to act as reducing agents. The entire properties and behavior of the NPs fluctuate by changing the morphology of NPs [23,24].

The antibacterial potential of the NPs alone, with plant extract and in synergy with antibiotics, was checked at different concentrations against both Gram positive bacteria (*S. aureus* and *S. pyrogenes*) and Gram negative bacteria (*E. coli* and *P. aeruginosa*). It was observed that plant extract alone and chemically synthesized NPs had fewer antibacterial effects compared to green synthesized NPs. Antibacterial and synergistic results revealed that green synthesized ZnO and TiO_2_ NPs had excellent inhibitory effects against both Gram positive and negative bacteria, which were increased by increasing the concentration of NPs. Previously, Liaqat et al. [38] reported that biogenically synthesized NPs exhibited strong antibacterial activity against a range of bacteria such as *Bacillus subtilis*, *Klebsiella pneumonia*, *Pseudomonas aeruginosa*, and *Proteus mirabilis*. Thus, they may be used in the surface coating of food packages to prevent bacterial contamination. There are several possible explainations for the bactericidal effect of ZnO and TiO_2_ particles. NPs exhibit antimicrobial activity due to their strong oxidizing property following exposure to sunlight or UV-light. For example, irradiated TiO_2_ particles particularly target the microbial surface as a primary site during initial oxidative attack.

In our study, ZnO and TiO_2_ NPs caused increased ZOI along with antibiotics (imipenem and ciprofloxacin). Using the imipenem and ciprofloxacin discs in combination with the NPs makes even ciprofloxacin susceptible. Similar results were also depicted by Rashid et al. [39], who reported that bacteria became sensitive to an antibiotic (ciprofloxacin) when combined with biosynthesized ZnO NPs, while the antibiotic (meropenem) exhibited improved behavior. It was also observed that the antibacterial activity of ZnO and TiO_2_ NPs was time dependent and takes effects gradually. The results showed that ZnO NPs were more efficient compared to TiO_2_. Similarly, Rajashekara et al. [40] synthesized ZnO NPs using *Calotropis gigantea* leaf extract and observed strong antibacterial activity against both Gram positive and negative bacteria. Our study is also in accordance with Rashid et al. [39], who examined the antibacterial activity of ZnO NPs alone and in combination with the β-lactam antibiotics viz. imipenem and ciprofloxacin. They discovered that ZnO NPs have strong antibacterial activity and a strong synergistic impact with β-lactam antibiotics. ZnO has excellent potential for use in medicine [38,41]. According to the previous literature [42], among the different NPs, TiO_2_ NPs are comparatively the most analyzed for their photocatalytic antibacterial activity.

Next, we determined the antioxidant potential and toxicity evaluation of ZnO and TiO_2_ NPs. DPPH free radical scavenging is an accepted mechanism for screening the antioxidant activity of plant extracts. It is the most widely documented method where antioxidants react with DPPH and convert it to yellow-colored diphenylpicrylhydrazine. The extent of color fading indirectly proves the radical-scavenging capacity of the antioxidant. The results showed excellent antioxidant activity because they can scavenge DPPH radicals. Antioxidants are substances that prevent and slow the lipid oxidation of other biomolecules and prevent the cell from damage in the process [43]. The antioxidant activity of NPs was tested by scavenging (DPPH) free radicals at various doses of NPs (10, 20, 30, and 40 mg/mL) and time intervals. ZnO NPs showed more radical scavenging activity compared to TiO_2_. The scavenging activity of DPPH radicals was found to be increased with the increased concentration of NPs. ZnO and TiO_2_ NPs antioxidant activity was observed to be the highest at 40 mg/mL concentration. A very similar study by Rajeshkumar et al. [44] supported our results of concentration-dependent increases in the antioxidant activity of NPs.

Kidney is a target organ in acute or chronic toxicity assessment for its capacity to filter, reabsorb, and concentrate divalent ions [45]. The toxicity evaluation of ZnO and TiO_2_ NPs was performed by a histological study of the kidney of male albino mice. After exposure to NPs (ZnO and TiO_2_), it was observed that small-sized NPs damaged the structure of the kidney. The toxicity level of green synthesized NPs was dose-dependent. A high level of toxicity was observed in the kidney of mice that were treated with a high dose (300 mg/kg) compared to mice treated with a low dose (100 mg/kg). No structural damage in the kidney of the control group was recorded. The histological changes observed in mice kidney following exposure to high dose/s were degeneration, swelling, and shrinkage of glomeruli. Edema formation and enlarged capsular space in the renal cortex were increased in the mice treated with a high dose (300 mg/kg). A similar study was conducted by Noori et al. [30] which supports our results. They investigated the effect of ZnO NPs on the kidneys of mice. It was found that ZnO NPs caused histologic changes in the kidneys, leading to the accumulation of inflammatory cells in glomerular capillaries and the degeneration of proximal as well as distal tubules. Interestingly, the authors found a short-term effect of ZnO NPs on renal function and reported that the gradual elimination of NPs uptake by kidneys led to the disappearance of toxic effects after a month.

## 5. Conclusions

The present research work was designed to determine the antibacterial, antioxidant, and toxicity impact of green synthesized ZnO and TiO_2_ NPs. ZnO and TiO_2_ NPs showed strong antibacterial and synergistic potential against both Gram negative and positive bacteria. However, the antibacterial effect of ZnO NPs was more promising compared to TiO_2_ NPs. Also, TiO_2_ NPs showed strong antioxidant activity compared to ZnO, and their radical scavenging activity increased in a concentration-dependent manner. Histological examination revealed that ZnO NPs had adverse effects on the kidney of albino mice compared to TiO_2_ NPs. Overall, the results showed that plant-based ZnO and TiO_2_ NPs displayed better antibacterial and antioxidant properties due to their biocompatibility. In brief, green syntheized NPs can be integrated in drug delivery systems and other bioactivities such as the food, feed, space, chemical, and cosmetics industries, provided that some unaddressed issues, including consistency in particle size and shape, reproducibility, and understanding of the mechanisms involved in producing metallic NPs using biological entities, are resolved. Further, the extrapolation of laboratory experiments to an industrial scale is highly important [46]. Also, more attention is required on the potential toxicity of green synthesized NPs, their route of administration, and their inclusion in commercial products [47] before their use in human consumer products.

## Figures and Tables

**Table 1:** Antibacterial activity and synergistic effects of *C. procera* synthesized ZnO NPs by disc diffusion method.

Strains			Zone of Inhibition (mm)							
			Concentration of *C. procera* Synthesized ZnO NPs (mg/mL)				ZnO NPs (20 mg/mL) + Antibiotics			
	Plant Extract	Chemically Synthesized ZnO NPs	10	20	30	40	IMP	ZnO + IMP	CIP	ZnO + CIP
*S. aureus*	6 ± 0.5	7 ± 0.4	8 ± 0.5	12 ± 0.5	15 ± 0.2	17 ± 0.2	25 ± 0.5	31 ± 0.1	R	13 ± 0.1
*S. pyrogenes*	7 ± 0.3	5 ± 0.3	7 ± 0.3	10 ± 0.5	13 ± 0.5	16 ± 0.5	27 ± 0.1	30 ± 0.5	R	12 ± 0.5
*E. coli*	6.5 ± 0.5	6 ± 0.4	7 ± 0.5	10 ± 0.3	10 ± 0.1	12 ± 0.1	18 ± 0.5	24 ± 0.3	R	11 ± 0.1
*P. aeruginosa*	6 ± 0.2	7 ± 0.3	9 ± 0.2	10 ± 0.2	11 ± 0.5	13 ± 0.5	21 ± 0.2	27 ± 0.2	R	12 ± 0.2

**Table 2:** Antibacterial and synergistic effects of *C. procera* synthesized TiO_2_ NPs by disc diffusion method.

Strains			Zone of Inhibition in mm							
			Concentration of *C. procera* Synthesized TiO_2_ NPs (mg/mL)				TiO_2_ NPs (20 mg/mL) + Antibiotics			
	Plant Extract	Chemically Synthesized TiO_2_ NPs	10	20	30	40	IMP	TIO_2_ + IMP	CIP	TiO_2_ + CIP
*S. aureus*	7 ± 0.3	5 ± 0.3	10 ± 0.3	11 ± 0.2	12 ± 0.5	14 ± 0.5	25 ± 0.5	31 ± 0.3	R	13 ± 0.1
*S. pyrogenes*	6 ± 0.3	7 ± 0.3	9 ± 0.3	10 ± 0.5	11 ± 0.5	13 ± 0.5	27 ± 0.3	30 ± 0.5	R	12 ± 0.2
*E. coli*	6.5 ± 0.2	7 ± 0.4	6 ± 0.2	9 ± 0.2	10 ± 0.1	10 ± 0.1	18 ± 0.5	24 ± 0.5	R	11 ± 0.5
*P. aeruginosa*	7 ± 0.5	6 ± 0.4	8 ± 0.5	9 ± 0.2	9 ± 0.3	11 ± 0.2	21 ± 0.5	27 ± 0.2	R	12 ± 0.5

## Data Availability

All the data is available in the manuscript.
